# Modelling the dynamics of acute and chronic hepatitis B with optimal control

**DOI:** 10.1038/s41598-023-39582-9

**Published:** 2023-09-11

**Authors:** Tahir Khan, Fathalla A. Rihan, Hijaz Ahmad

**Affiliations:** 1 Department of Mathematical Sciences, College of Science, UAE University, 15551 Al-Ain, United Arab Emirates; 2 Department of Mathematics, Faculty of Science, Islamic University of Madinah, Medina, 42210 Saudi Arabia; 3 Near East University, Operational Research Center in Healthcare, TRNC Mersin 10, Nicosia, 99138 Turkey; 4 Department of Computer Science and Mathematics, Lebanese American University, Beirut, Lebanon

**Keywords:** Biological techniques, Mathematics and computing

## Abstract

This article examines hepatitis B dynamics under distinct infection phases and multiple transmissions. We formulate the epidemic problem based on the characteristics of the disease. It is shown that the epidemiological model is mathematically and biologically meaningful of its well-posedness (positivity, boundedness, and biologically feasible region). The reproductive number is then calculated to find the equilibria and the stability analysis of the epidemic model is performed. A backward bifurcation is also investigated in the proposed epidemic problem. With the help of two control measures (treatment and vaccination), we develop control strategies to minimize the infected population (acute and chronic). To solve the proposed control problem, we utilize Pontryagin’s Maximum Principle. Some simulations are conducted to illustrate the investigation of the analytical work and the effect of control analysis.

## Introduction

Hepatitis B, a non-cytopathic virus, causes inflammation of the liver. As the virus infects hepatocytes, it does not completely destroy the host cells. The immune system, however, responds by inflaming the liver^1^. When the virus enters the body, it contaminates the hepatocytes in the liver^2^. The main cause of hepatitis is the exposure of an individual to alcohol or drugs as well as bacterial infections^3^. Acute and chronic hepatitis are the two stages of this disease. During the first 180 days after exposure to the hepatitis B virus, the immune system may be able to remove the virus, resulting in a complete recovery. In some cases, however, the infection may grow and progress to the chronic stage of hepatitis B. Six months after infection, the infectious individual’s HBsAg will be positive. Most often, at this stage, no acute illness has been experienced. It has also been shown that liver scarring can lead to liver failure and liver cancer^4^. The virus can be transmitted via blood (sharing blades and razors, etc.) and vaginal and semen secretions^5,6^. Another major route of transmission, also called vertical transmission, is from the mother to the newborn^7^. According to the World Health Organization (WHO), there are millions of chronically infected individuals around the world, but only 93 million of them live in China (see^8,9^). However, Hepatitis B can be prevented with vaccines^10,11^.

Researchers have extensively used mathematical modeling of infectious diseases (e.g.,^12–20^). There is an extensive literature on the epidemiology of hepatitis B. Many biologists and mathematicians have studied the temporal dynamics of the disease; see, for instance,^21^. A simple mathematical model was developed by Anderson and co-authors to describe carries influence on hepatitis B virus transmission^22^. In 1996, Williams et al. presented a model to study hepatitis B dynamics in the United Kingdom /cite[williams1996transmission], whereas Medley et al. presented a model that predicted an eliminating mechanism for hepatitis B in New Zealand^23^. The vaccination program and its effectiveness have also been studied using a mathematical model^24^. An epidemic model has also been used to study a control analysis in^25^. Kamyad et al. proposed a different control strategy for hepatitis B in their paper^26^. Hepatitis B epidemic problems and vertical transmission were addressed by Onyango et al. in^27^. The time dynamics of hepatitis B in Xinjiang, China, were also investigated in^28^. Khan et al. have recently discussed epidemic models of hepatitis B dynamics by incorporating a variety of influential parameters. See citations^29–31^

It is important to know that the different phases of hepatitis B disease (acute and chronic) and their transmission routes (horizontal and vertical) all contribute to the spread of the infection since carriers do not experience symptoms and transmit the disease. To our knowledge, the current study investigates the impact of different phases of infected individuals and different transmission routes, which have not yet been considered together to formulate a hepatitis B virus model. A few control mechanisms are also outlined that may help eliminate the infection. The basic axioms of the problem are discussed in detail to illustrate the feasibility in both aspects, mathematically and biologically. Herein, we propose a novel epidemic model of hepatitis B dynamics under distinct infection phases and multiple transmissions. Two control measures with dependency on time, i.e., vaccination and treatment, are considered to describe an optimal control strategy. The objective is to reduce the infected proportion by vaccinating the susceptible class and treating the infected class at the cost of such control functions. A contrast to^31^, in which the optimal control policy calls for the isolation of infected and non-infected individuals, is that our optimal strategies do not rely on isolation or quarantine because quarantine and isolation are always governed by the relevant public health agencies and are not recommended by the WHO in cases of hepatitis B.

The outline of this paper is as follows. The proposed model with its properties is provided in “Problem formulation” section 2. Based on the next-generation matrix, we calculate the basic reproductive number in “Basic reproductive number” section 3. LaSalle’s invariance principle, linear stability, and geometrical approaches are used in “Existence of backward bifurcation” section 4 to analyze the dynamics of the proposed problem. Furthermore, we reveal the backward bifurcation analysis for the epidemic problem in “Stability analysis” section 5. We demonstrate the existence of our control problem, apply necessary optimality conditions in “Formulation of control problem” section 6, and illustrate all the theoretical findings by numerical simulations in “Numerical simulations” section 7. Some concluding remarks are presented in “Conclusions” section 8.

## Problem formulation

Based on disease transmission characteristics, an epidemic model is proposed to investigate hepatitis B virus transmission. There are four classes of host populations, symbolized by *N*(*t*): the susceptible class *S*(*t*), the acutely infected compartment *A*(*t*), the chronically ill class *B*(*t*), and the immunized/recovered class *R*(*t*). The following assumptions are made in our model.

$$a_1$$.
Each parameter, as well as the variable used in the proposed epidemic problem, is nonnegative.
$$a_2$$.
The vaccine for hepatitis B is very effective because it provides indefinite protection; therefore, the susceptible individuals, after being vaccinated successfully, lead to the recovered population.
$$a_3$$.
Both the acutely infected and chronically infected individuals will cause the infection to be susceptible, and by successful interaction, the susceptible will lead to the acute class.
$$a_4$$.
Natural death occurs in each model group, while death from disease only occurs in the chronic class.
$$a_5$$.
The portion of newborns with maternal infection leads to *B*(*t*).

**Figure 1:**
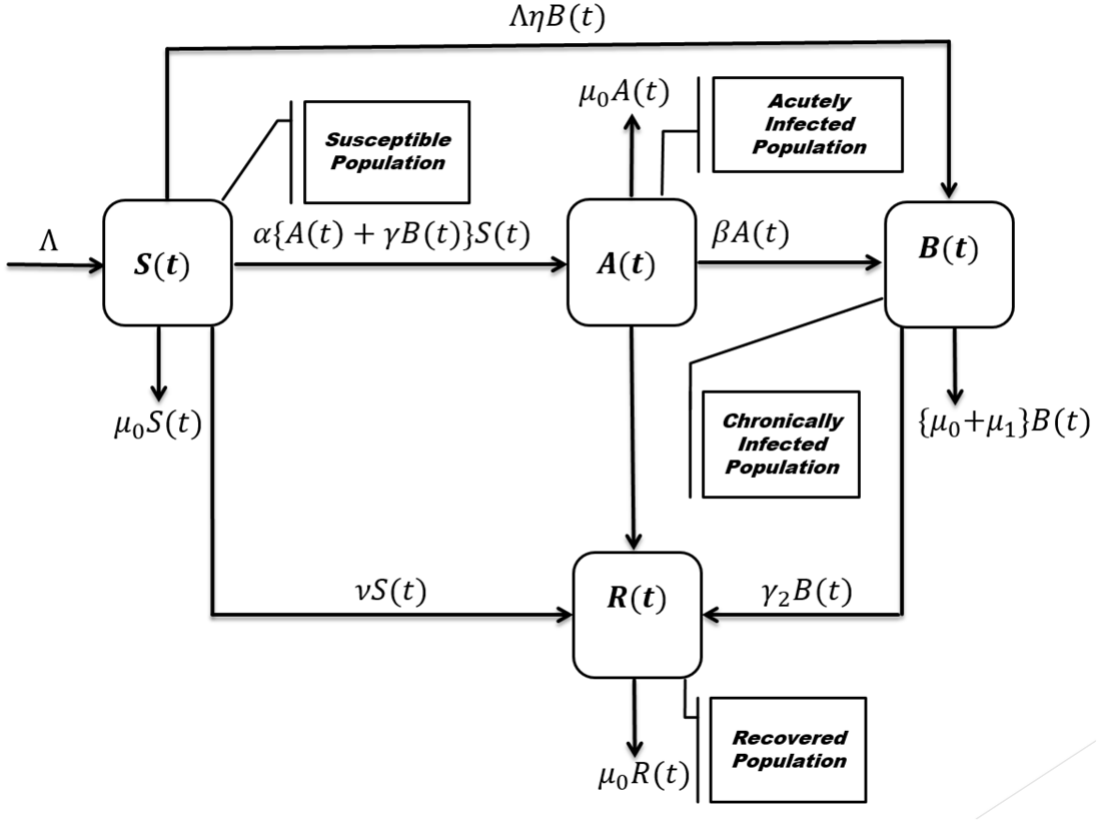
The schematic diagram for the transmission of the disease.

The schematic disease transmission process is demonstrated by Fig. 1. By grouping all of the above assumptions, a system of autonomous differential equations can be derived that describes the complete model

1 $$\begin{aligned} \begin{aligned} \frac{dS(t)}{dt}&=\left\{ 1-\eta B(t)\right\} \Lambda -\left\{ v+\mu _0\right\} S(t)-\left\{ A(t)+\gamma B(t)\right\} \alpha S(t),\\ \frac{dA(t)}{dt}&= \alpha S(t)A(t)+\gamma \alpha S(t)B(t)-\left\{ \gamma _1+\beta +\mu _0\right\} A(t),\\ \frac{dB(t)}{dt}&=\beta A(t)-\left\{ \mu _1+\gamma _2+\mu _0\right\} B(t)+\eta \Lambda B(t),\\ \frac{dR(t)}{dt}&=\gamma _2B(t)-\mu _0R(t)+\gamma _1 A(t)+vS(t). \end{aligned} \end{aligned}$$

Investigation of the model (1) is subject to the initial sizes of compartments

2 $$\begin{aligned} S(0)>0,~A(0)\ge 0,~B(0)\ge 0,~R(0)>0. \end{aligned}$$

The parameter $\Lambda$ in (1) is the rate of newborns, *v* is the vaccination parameter, and $\eta$ is the maternally infected rate. The symbol $\ gamma$ denotes the reduced transmission rate, and $\mu _0$ illustrates the proportion of natural death. Similarly, $\mu _1$ is the portion of deaths that occurs due to the disease. We represent the contact parameter by $\alpha$ and the recovery rate from the acute class by $\gamma _1$. Moreover, $\gamma _2$ symbolizes the recovery in chronically infected population, and $\beta$ is the proportion of those who move from acute class to chronic one.

First, we prove the well-posedness by illustrating the following results.

### Proposition 2.1

(Existence and uniqueness) The proposed epidemiological model (1) with initial conditions described by Eq. (2) possesses a unique solution.

### Proof

To determine that the model (1) possesses a unique solution, we follow the methodology given in^32^ and define the vector field of the proposed model $H:(-\infty ,\infty )\times \mathbb {R}^{4}\rightarrow \mathbb {R}^{4}$ as

3 $$\begin{aligned} H(S,A,B,R)=\left( \begin{array}{c} \left( 1-\eta B\right) \Lambda -\left( q_1+\psi \right) S \\ \psi S-q_2A \\ \eta \Lambda B-q_3B+\beta A \\ \gamma _1 A+ \gamma _2B-\mu _0R+vS \\ \end{array} \right) , \end{aligned}$$

where

4 $$\begin{aligned} q_1=v+\mu _0, \quad q_2=\mu _0+\gamma _1+\beta , \quad q_3=\mu _0+\gamma _2+\mu _1,\quad \psi =\alpha A+\gamma \alpha B. \end{aligned}$$

The right-hand side of the Eq. (3) implies that the function *H* is continuous and therefore ensures the existence of solution (*S*, *A*, *B*, *R*) over an interval $[0,\infty )$. In addition, calculating the derivative of *H* with respect to the model state variables gives the Jacobian matrix as given by

5 $$\begin{aligned} DH=\left( \begin{array}{cccc} \left( q_1+\psi \right) &{} -\alpha S &{} -\eta \Lambda -\gamma \alpha S &{} 0 \\ \psi &{} \alpha S-q_2 &{} \gamma \alpha S &{} 0 \\ 0 &{} \beta &{} \eta \Lambda -q_3 &{} 0 \\ v &{} \gamma _1 &{} \gamma _2 &{} -\mu _0 \\ \end{array} \right) . \end{aligned}$$

Since, *DH* is continuous over $\mathbb {R}^4$ and thus *H* is locally Lipschitz continuous on $(-\infty ,\infty )\times \mathbb {R}^{4}$, therefore, the model solution (*S*, *A*, *B*, *R*) is uniquely determined on the interval $[0,\infty )$. $\square$

### Proposition 2.2

(Positivity of solution) Let the solution to the problem (1)–(2) be symbolized by (*S*, *A*, *B*, *R*), whenever exists, then it is positive for all *t* greater than zero.

### Proof

Obviously, right-side functions in the system (1) satisfy the conditions of differentiability, implying the existence of a unique maximal solution for any associated Cauchy problem. Thus, the first equation of system (1) takes the form

6 $$\begin{aligned} \frac{dS}{dt}=\Lambda (1-\eta B)-\varphi S, \end{aligned}$$

where $\varphi =q_1+\psi$. The solution of (6) looks like

7 $$\begin{aligned} S(t)= & {} S(0)\exp \left\{ -\int ^{t}_{0}\varphi (x)dx\right\} \nonumber \\{} & {} +\exp \left\{ -\int ^{t}_{0}\varphi (x)dx\right\} \left[ \int ^{t}_{0} \Lambda (1-\eta B) \exp \left\{ \int ^{\ell }_{0}\varphi (u)du\right\} dx\right] . \end{aligned}$$

Following the same steps, the model, second equation can be re-casted as

$$\begin{aligned} \frac{dA}{dt}\ge -q_2A, \end{aligned}$$

which leads to

8 $$\begin{aligned} A(t)=A(0)\exp \left( -q_2t\right) . \end{aligned}$$

Similarly, the last two equations of the epidemiological model (1) can be re-written as

$$\begin{aligned} \frac{dB}{dt}\ge -q_3B \quad \text {and}\quad \frac{dR}{dt}\ge -\mu _0R. \end{aligned}$$

Integrating, we then obtain

9 $$\begin{aligned} B(t)\ge B(0)\exp \left( -q_3t\right) \quad \text {and}\quad R(t)\ge R(0)\exp \left( -\mu _0t\right) . \end{aligned}$$

Thus, from the above Eqs. (7)–(9), it could be observed that all the state variables of the proposed epidemiological model satisfying the initial conditions remain non-negative. $\square$

### Proposition 2.3

(Bounded-ness of solution) Solution of the problem (1)–(2) is bounded.

### Proof

Let

10 $$\begin{aligned} N=S+A+B+R. \end{aligned}$$

Taking the temporal derivatives of this equation and exploiting values from model (1), one may obtain

11 $$\begin{aligned} \frac{dN}{dt}+\mu _0N=\Lambda -\mu _1B. \end{aligned}$$

Since by assumption $\mu _0$ is a positive parameter and $B\ge 0$. Consequently we may write $\frac{dN}{dt}+\mu _0N\le \Lambda$. Solution of this equation subject to the initial conditions (2) gives

12 $$\begin{aligned} 0<N\le \frac{\Lambda }{\mu _0}+\left( N(0)-\frac{\Lambda }{\mu _0}\right) \exp \left( {-\mu _0t}\right) . \end{aligned}$$

It is obvious that whenever $t\rightarrow \infty$, the last equation yields $0<N\le \frac{\Lambda }{\mu _0}$. $\square$

### Proposition 2.4

(Positively invariant set) Let *N* be the total population as given in (10), then the feasible region represented by

13 $$\begin{aligned} \Delta =\left\{ (S, A, B, R)\in \mathbb {R}^4_+:0<N\le \frac{\Lambda }{\mu _0}\right\} , \end{aligned}$$

is invariant positively and attracting for the proposed epidemiological model (1).

### Proof

Since, $N=S+A+B+R$, if $N(0)\le \frac{\Lambda }{\mu _0}$, then clearly Eq. (12) implies that $N(t)\le \frac{\Lambda }{\mu _0}$. But on the other hand, on a contrary basis, if $N(0) \ge \frac{\Lambda }{\mu _0}$, then either the total population *N*(*t*) converge to $\frac{\Lambda }{\mu _0}$ as *t* increases without bound or the solution trajectories enter the feasible region $\Delta$ within finite time, which implies that all the state variables initiated in $\mathbb {R}^4_+$ enter $\Delta$ or converge $\frac{\Lambda }{\mu _0}$ eventually. $\square$

## Basic reproductive number

There are two possible non-trivial equilibrium points of model (1), namely, the endemic and disease-free states. The disease-free state is represented by $E_1$ and is calculated as $E_1=(S_1, 0, 0, R_1)$, such that

14 $$\begin{aligned} S_1=\frac{\Lambda }{q_1},~R_1=\frac{\Lambda v}{\mu _0 q_1}. \end{aligned}$$

We use this state to calculate the so-called basic reproductive quantity, $R_0$, which describes the average number of secondary infectious created by an index case, i.e. when an infective is presented into a susceptible population so the secondary infections are produced during its total infection age^33^. The reproductive number $R_0$ is then conveniently used to characterize the endemic equilibrium. Let us assume that *J* represents the linearized matrix of the system (1). Direct calculations show that the matrix *J* has the form

$$\begin{aligned} J=\left( \begin{array}{cccc} -q_1-\alpha A-\gamma \alpha B &{} -\alpha S &{} -\Lambda \eta -\gamma \alpha S &{} 0 \\ \alpha A+\gamma \alpha B &{} \alpha S-q_2 &{} \gamma \alpha S &{} 0 \\ 0 &{} \beta &{} -q_3 &{} 0\\ v &{} \gamma _1 &{} \gamma _2 &{} -\mu _0\\ \end{array} \right) , \end{aligned}$$

with $q_1$, $q_2$ and $q_3$ as given in (4). We follow Watmough and Driessche^34^ to determine the threshold number of the epidemiological model that is under consideration. By assuming $X = \left( A(t),B(t)\right) ^T$, one can write from (1) that

15 $$\begin{aligned} \frac{dX}{dt}=\bar{F}-\bar{V}, \end{aligned}$$

where $\bar{F}$ and $\bar{V}$, in the above equation, are defined as

$$\begin{aligned} \bar{F}=\left[ \begin{array}{c} \alpha AS+\gamma \alpha SB\\ 0 \\ \end{array} \right] ,\quad \bar{V}=\left[ \begin{array}{c} q_2A\\ -\beta A+q_3B\\ \end{array} \right] . \end{aligned}$$

The Jacobian or the linearized matrices of the above-defined $\bar{F}$ and $\bar{V}$ at the infection-free state (14) are respectively calculated as

$$\begin{aligned} F =\left[ \begin{array}{cc} \alpha S_1 \ {} &{} \gamma \alpha S_1\\ 0 \ {} &{} 0 \\ \end{array} \right] , \quad V =\left[ \begin{array}{cc} q_2\ &{} 0 \\ -\beta \ &{} q_3 \\ \end{array} \right] . \end{aligned}$$

The threshold quantity, $R_0$ is given by the largest eigenvalue of the matrix $F V^{-1}$. That is, $R_0=\rho (FV^{-1})$. We deduced that

16 $$\begin{aligned} R_0=R_{01}+R_{02},~\text {where}~R_{01}=\frac{\alpha \Lambda }{q_1q_2},~R_{02}=\frac{\alpha \gamma \beta \Lambda }{q_1q_2q_3}. \end{aligned}$$

Now we find the endemic-state of the model using (1) and (16), we obtain

17 $$\begin{aligned} {\left\{ \begin{array}{ll} \begin{aligned} E^{*}_2&{}=\left( S^{*}, A^{*}, B^{*},R^{*}\right) , \quad S^{*} =\frac{1}{q_1}\big \{\Lambda (1-\eta B^{*})-q_2A^{*}\big \},\\ \quad A^{*}&{}=\frac{q_1q_2q_3^2\{R_0-1\}}{\Lambda \eta \beta \{\alpha q_3+\gamma \alpha \beta \}+\alpha q_2q_3^2+\gamma \alpha \beta q_2q_3},\\ \quad B^{*}&{}=\frac{ q_1q_2q_3\beta \{R_0-1\}}{\alpha \{q_3+\gamma \beta \}\{\eta \beta \Lambda +q_2q_3\}},\\ \quad R^{*}&{}=\frac{1}{\mu _0}\big \{\gamma _1A^{*}+\gamma _2B^{*}+vS^{*}\big \}. \end{aligned} \end{array}\right. } \end{aligned}$$

The characterization of the occurrence of the no-infection state (14) and of the disease-endemic state (17) is investigated in “Existence of backward bifurcation” section 4, while in “Stability analysis” section 5 we investigate their global analysis.

## Existence of backward bifurcation

In epidemiological models, one of the necessary conditions to control the infection is $R_0<1$. In contrast, this condition may not always be sufficient, owing to backward bifurcation, i.e., a stable endemic state co-exists with a stable infection-free state whenever $R_0<1$. It is a common phenomenon in epidemiological models^35^. In this case, disease control depends upon the various sub-populations sizes of the epidemic problem. To investigate the existence of bifurcation, we suppose that at least one of the infected groups in system (1) is nonzero. In this situation, the solution of our proposed model (1) around steady state yields

$$\begin{aligned} S^{*}=\frac{1}{q_1}\left\{ \Lambda \{1-\eta B^{*}\}-q_2A^{*}\right\} , \quad A^{*} = \frac{1}{\beta }q_3B^{*}. \end{aligned}$$

For $B^{*}\ne 0$, we insert $A^{*}$ and $S^{*}$ in system (1) around steady state, and utilizing (4), we obtain the following equations

18 $$\begin{aligned} \psi (B)= & {} aB^{2}+bB+c,\ \text {where}\\ a= & {} \left\{ \alpha \Lambda \eta \beta q_3+\alpha q_2q_3^2+\gamma \alpha \beta ^2\eta \Lambda +\gamma \alpha q_2q_3\beta \right\} /\beta q_1,\nonumber \\ b= & {} \left\{ \beta \mu _0\Lambda +q_1q_2q_3(1-R_{01}-R_{02})\right\} /q_1\beta ,~\quad c=0.\nonumber \end{aligned}$$

It could be noted from the last relation that whenever the condition of $R_0<1$ holds, then *b* and *c* are non-negative. Also if $R_0>1$ then $b<0$. Clearly, $a>0$, so a positive solution of equation (18) exists, which depends on the signs of *b*, proving that the equilibrium continuously depends on the threshold quantity. Moreover equation (18) implies that

$$\begin{aligned} B_1=\frac{-b+\sqrt{b^2-4ac}}{2a}, \quad B_2=\frac{-b-\sqrt{b^2-4ac}}{2a}. \end{aligned}$$

For distinct ranges of the parameters, we state the underlying result.

### Theorem 4.1

The considered epidemic problem (1) has:

(i) a unique endemic state in the biologically reasonable region $\Delta$ (13) whenever *b* is negative and $R_0>1$;
(ii) a unique endemic state in $\Delta$ (13) if $b=0$;
(iii) two endemic steady states in $\Delta$ (13) whenever $b>0$.

It could be noted in the epidemiological models that one of the classical requirements for disease elimination is $R_0<1$, while this is not sufficient^35^. Thus the condition $R_0<1$ is necessary for the control of hepatitis B but is not sufficient. Moreover, the backward bifurcation’s presence in the model states that elimination of hepatitis B in case of $R_0<1$ depends on sub-populations of the model, and whenever $R_0=1$, we have described the following.

### Lemma 4.2

The existence of backward bifurcation for the model (1) depends on the value of $R_0$ and exists whenever $R_0=1$, while experiences backward bifurcation in case condition (*iii*) of Theorem 4.1 holds.

## Stability analysis

We now demonstrate the global analysis of the problem (1) at both the non-trivial equilibria. For the global properties around disease-free state (14), we use the classical Lyapunov function theory, while at disease-endemic state (17), we use the geometrical approach.

### Theorem 5.1

(Global stability of (1) at $E_1$ (14)) The proposed system (1) is stable globally asymptotically at $E_1$ (14) whenever $R_0<1$ and $S\ge S_1$. Otherwise, (1) is unstable.

### Proof

Let $h_1>0$, $h_2>0$ and $h_3>0$ be constants to be determined later. Consider a function of the form

$$\begin{aligned} \ F(t)=h_1(S-S_1)+h_2A+h_3B. \end{aligned}$$

The temporal differentiation of this equation, along with values from system (1), gives

19 $$\begin{aligned} \frac{dF}{dt}= & {} h_1\left\{ (1-\eta B)\Lambda -\alpha A S-\gamma \alpha BS-q_1S\right\} \nonumber \\{} & {} +h_2\left\{ \alpha AS-q_2A+\gamma \alpha BS\right\} + h_3\left\{ A\beta -Bq_3\right\} . \end{aligned}$$

Let us assume $h_1=h_2=q_1$, and $h_3=\frac{\alpha \beta \Lambda \gamma }{q_3}$. Further, from our previous calculations have $S_1=\frac{\Lambda }{q_1}$. Then from the last equation, we have

$$\begin{aligned} \frac{dF}{dt}&= q_1\left\{ (q_1S_1-\eta \Lambda B-\alpha AS-\gamma \alpha BS-q_1S)\right\} \\{} & {} +q_1\left\{ (\alpha S A+\gamma \alpha S B- q_2 A)\right\} +\frac{\alpha \beta \gamma \Lambda }{q_3}\left\{ \beta A-q_3B\right\} . \end{aligned}$$

Simplification of the above equation leads to

$$\begin{aligned} \frac{dF}{dt}=-q_1^2\left\{ S-S_1\right\} -\left\{ q_1q_2(1-R_{02})\right\} A -\left\{ q_1\Lambda \eta +\alpha \Lambda \gamma \right\} B. \end{aligned}$$

Thus, when $R_0<1$, we have $0<R_{01}<1$ and $0<R_{02}<1$, then $\frac{dF}{dt}<0$. Also, $\frac{dF}{dt}=0$ if $S=S_1$, and $B=A=0$. Hence, the principle of LaSalle’s^36,37^ reveals that (14) is stable globally asymptotically. $\square$

### Theorem 5.2

(Global stability of (1) at $E_2^{*}$ (17)) If $R_0$ assumes values greater than 1, the disease presence state $E^{*}_2=(S^{*}, A^{*}, B^{*}, R^{*})$ of model (1) is globally stable. Endemic state of (1) is unstable whenever $R_0>1$ does not hold.

### Proof

Reducing model (1) by removing *R*(*t*) with the fact that it can be derived from the relation of the total populace, i.e., $N=A+S+B+R$, which implies that $R=N-S-A-B$. So without losing generality, it is enough to discuss the dynamics of the reduced model for the original. Thus, let $J_2$ is the Jacobian while $J^{\mid 2\mid }_2$ is the second order compound matrix of the proposed model (1), then

$$\begin{aligned} \begin{aligned} J_2&=\left( \begin{array}{ccc} -\rho _{11} &{} -\rho _{12} &{} -\rho _{13} \\ \rho _{21} &{} -\rho _{22} &{} \rho _{23} \\ \rho _{31} &{} \rho _{32} &{} -\rho _{33} \\ \end{array} \right) ,\\ J_2^{\mid 2\mid }&=\left( \begin{array}{ccc} -(\rho _{11}+\rho _{22}) &{} \rho _{23} &{} -\rho _{13}\\ \rho _{32} &{} -(\rho _{11}+\rho _{33}) &{} \rho _{12}\\ -\rho _{31} &{} \rho _{21} &{} -(\rho _{22}+\rho _{33})\\ \end{array} \right) , \end{aligned} \end{aligned}$$

where

$$\begin{aligned} \begin{aligned} \rho _{11}&=q_1+\alpha A+\gamma \alpha B,\quad \rho _{12}=\alpha S,\quad \rho _{13}=\Lambda \eta +\gamma \alpha S,\\ \rho _{21}&=\alpha A+\gamma \alpha B,\quad \rho _{22}=q_2-\alpha S,\quad \rho _{23}=\gamma \alpha S,\\ \rho _{31}&=0,\quad \rho _{32}=\beta ,\quad \rho _{33}=q_3. \end{aligned} \end{aligned}$$

We consider a function in the form of $P=P(S,A,B)=\textrm{diag}\left\{ S/A,S/A,S/A\right\}$, then taking its inverse and differentiating with respect to *t*, i.e. $P_f(\chi )$, we have

$$\begin{aligned} P_f={\text{diag}}\left\{ \frac{\dot{S}}{A}-\frac{\dot{A}S}{A.A}, \frac{\dot{S}}{A}-\frac{\dot{A}S}{A.A}, \frac{\dot{S}}{A}-\frac{\dot{A}S}{A.A}\right\} . \end{aligned}$$

Implying that $P_fP^{-1}=\text{diag}\left\{ -\dot{A}/A+\dot{S}/S,-\dot{A}/A,-\dot{A}/A\right\}$ and $J^{\mid 2\mid }_2=PJ^{\mid 2\mid }_2P^{-1}$. So

$$\begin{aligned} B=PJ^{\mid 2\mid }_2P^{-1}+P_fP^{-1}, \end{aligned}$$

implies that

$$\begin{aligned} B=\left( \begin{array}{cc} B_{11} &{} B_{12}\\ B_{21} &{} B_{22} \end{array} \right) , \end{aligned}$$

where

$$\begin{aligned}B_{11}&=\frac{\dot{S}}{S}-\frac{\dot{A}}{A}-q_1-q_2-\alpha A-\gamma \alpha B-\alpha S,\\ B_{12}&=\left( \begin{array}{cc} \alpha S &{} \Lambda \eta +\gamma \alpha S\\ \end{array} \right) , \quad B_{21}=\left( \begin{array}{c} \beta \\ 0 \end{array} \right) , \\ B_{22}&=\left( \begin{array}{cc} \frac{\dot{S}}{S}-\frac{\dot{A}}{A}-q_1-q_3-\alpha A-\gamma \alpha B &{} -\alpha S \\ \alpha A+\gamma \alpha B &{} \frac{\dot{S}}{S}-\frac{\dot{A}}{A}-q_2-q_3+\alpha S \\ \end{array} \right)\end{aligned} .$$

Let $$(b_1,b_2,b_3)\in \mathbb {R}^{3}$$ then

$$\begin{aligned} \Vert b_1,b_2,b_3\Vert =\max \{\Vert b_1\Vert ,\Vert b_2\Vert +\Vert b_3\Vert \}. \end{aligned}$$

Let $\ell (B)$ is the Lozinski measure^38^ with respect to the above equation, then it becomes

$$\begin{aligned} \ell (B) \le \sup \left\{ g_2,g_1\} =\sup \{\Vert B_{12}\Vert +\ell (B_{11}),\quad \Vert B_{21}+\ell (B_{22})\Vert \right\} , \end{aligned}$$

where $g_i=\Vert B_{ij}+\ell (B_{ii})\Vert$ for $i,j=1,2$ and $i\ne j$, so then $g_1$ and $g_2$ are defined by

$$\begin{aligned} g_1=\Vert B_{12}+\Vert \ell (B_{11}), \end{aligned}$$

and

$$\begin{aligned} g_2=\Vert B_{21}\Vert +\ell (B_{22}). \end{aligned}$$

In the above last two equations

$$\begin{aligned} \begin{aligned} \ell (B_{11})&=\frac{\dot{S}}{S}-\frac{\dot{A}}{A}-q_1-q_2-\alpha A-\gamma \alpha B-\alpha S,\\ \ell (B_{22})&=\max \left\{ \frac{\dot{S}}{S}-\frac{\dot{A}}{A}-q_3-q_1, \frac{\dot{S}}{S}-\frac{\dot{A}}{A}-q_2-q_3\right\} {,}\\&=\frac{\dot{S}}{S}-\frac{\dot{A}}{A}-\mu _0-\min \{v,\gamma _1+\beta -q_3\}, \end{aligned} \end{aligned}$$

$\Vert B_{12}\Vert =\Lambda \eta +\gamma \alpha S$ and $\Vert B_{21}\Vert =\max \{\beta ,0\}=\beta$. Consequently $g_1$ and $g_2$ can be written as

$$\begin{aligned} \begin{aligned} g_1&=\frac{\dot{S}}{S}-\frac{\dot{A}}{A}-q_1-q_2-\alpha A-\gamma \alpha B-\alpha (1-\gamma )S+\Lambda \eta ,\\ g_2&=\frac{\dot{S}}{S}-\frac{\dot{A}}{A}-q_3-\min \{\gamma _1+\beta ,v\}, \end{aligned} \end{aligned}$$

gives

$$\begin{aligned} \begin{aligned} \ell (B)&\le \sup \{g_1,g_2\}\\&= \frac{\dot{S}}{S}-\frac{\dot{A}}{A}-2\mu _0 -\min \bigg \{v+\gamma _1+\beta +\alpha A+\gamma \alpha B+\alpha (1-\gamma )S,\gamma _2+\mu _1\\&+\min \{\beta +\gamma _1,v\}-\beta -\eta \Lambda \bigg \}. \end{aligned} \end{aligned}$$

From here, we can write $\ell (B)\le \frac{\dot{S}}{S}-2\mu _0$. The application of integration for $\ell (B)$ in [0, *t*] with $\lim$ as *t* approaches $\infty$ gives

$$\begin{aligned} \lim _{t\rightarrow \infty }\sup \sup \bar{q}= \frac{1}{t} \int ^{t}_{0}\ell (B)dt\le -2\mu _0<0{,} \end{aligned}$$

is negative, which proves the conclusion. $\square$

According to the stability analysis of the model, the reproductive number is an important parameter controlling disease dynamics. In the following subsection, we will discuss how parameters affect reproductive numbers.

### Influential parameters and its relative impact

For the purpose of creating an effective control mechanism for disease elimination, we conduct a sensitivity analysis of the model parameters in order to uncover the most influential parameters that highly affect the basic reproductive number. By using the formula given below, we can calculate the sensitivity indices and determine how these parameters affect the basic reproductive number.

20 $$\begin{aligned} H_{\phi }^{R_0}=\frac{\phi }{R_0}\frac{\partial R_0}{\partial \phi }, \end{aligned}$$

where $\phi$ is any epidemic parameter of the proposed epidemiological model associated with the reproductive number $R_0$. By following the formula (20), we obtain the sensitivity indices of the proposed model parameters as

21 $$\begin{aligned} \begin{aligned} H_{\alpha }^{R_0}&=1,\quad H_{v}^{R_0}=-\frac{v}{v+\mu _0},\quad H_{\gamma _1}^{R_0}=-\frac{\gamma _1}{\mu _0+\gamma _1+\beta },\\ H_{\gamma _2}^{R_0}&=-\frac{\gamma \gamma _2\beta }{(\mu _0+\gamma _2+\mu _1)(\mu _0+\gamma _2+\mu _1+\gamma \beta )}. \end{aligned} \end{aligned}$$

Clearly, the normalized sensitivity indices of the parameters $\alpha$, *v*, $\gamma _1$ and $\gamma _2$ show that $\alpha$ is positively correlated with the reproductive quantity, while *v*, $\gamma _1$ and $\gamma _2$ are negatively correlated. An increase in the values positively correlated results increase in the value of the reproductive number. But on, the value of the reproductive number will be reduced whenever the value of the negatively correlated parameters increases. Hence, control efforts should be formulated by taking suitable control measures to reduce the burden of hepatitis B virus transmission.

## Formulation of control problem

The theory of optimization is a prominent tool and is used frequently in the dynamics of infectious epidemiology. With the help of this, we can formulate strategies for the minimization of various kinds of infections. We follow the approach as presented by Gul et al. and others (see,^39–43^) to set up a control problem for the reduction of the hepatitis B infection. We propose a control mechanism to minimize the burden of hepatitis B virus transmission by utilizing two control measures (vaccination and treatment) because the normalized sensitivity indices of *v*, $\gamma _1$, and $\gamma _2$ have an inverse relationship with reproduction quantity. The aim is to vaccinate susceptible (control $u_1(t)$), and the treatment of infected (control $u_2(t)$). This is in contrast with^31^, where an inconsistent control problem was developed and consequently solved. Indeed, in^31^, the authors vaccinate all the infected individuals at the same rate as they vaccinate the susceptible. This seems not to be coherent with medical practice. In our case, the control system is obtained from (1) by placing two control measures already mentioned $u_1(t)$ and $u_2(t)$ with the description to vaccinate *S* as well as treatment of *A* and *B*. This implies that system (1) becomes a specific case of the proposed control problem whenever $u_1(t) \equiv v$ (vaccination with constant rate) and $u_2(t) \equiv 0$ (when there is no treatment). Thus, the control problem takes the form

22 $$\begin{aligned} J(u_1,u_2)=\int _0^T \left\{ w_1 A+w_2 B +\frac{1}{2}\left( w_3 u_1^2 + w_4 u_2^2\right) \right\} dt, \end{aligned}$$

subject to the problem

23 $$\begin{aligned} \begin{aligned} \frac{dS(t)}{dt}&= \left\{ 1-\eta B(t)\right\} \Lambda -\alpha S(t) A(t)-\gamma \alpha S(t) B(t)-\left\{ \mu _0+u_1(t)\right\} S(t),\\ \frac{dA(t)}{dt}&= \alpha S(t) A(t)+\gamma \alpha S(t) B(t)-\left\{ u_2(t)+\mu _0+\gamma _1+\beta \right\} A(t),\\ \frac{dB(t)}{dt}&= -\left\{ \mu _0-\Lambda \eta +\gamma _2+u_2(t)+\mu _1\right\} B(t)+\beta A(t),\\ \frac{dR(t)}{dt}&= \gamma _1 A(t)+\gamma _2B(t)+ u_1(t)S(t)+\left\{ B(t)+A(t)\right\} u_2(t)-\mu _0R(t), \end{aligned} \end{aligned}$$

In (22), $w_1$ and $w_2$ describe the relative weight constants of acute and chronic individuals, respectively. Also, the constants $w_3,~w_4\ge 0$ measure the associated costs of vaccination and treatment, respectively. It could be illustrated from (22) that the control problem has a clear purpose, namely, to reduce the ratio of *A* and *B* by implementing the control measures costs $u_1(t)$ and $u_2(t)$. However, it is not our objective to reduce or increase the number of susceptible, as inconsistently proposed in^31^. Indeed, our goal is to determine the control functions $(u_1^{*},u_2^{*})$ like

24 $$\begin{aligned} J(u_1^{*},u_2^{*})=\min \left\{ J(u_1,u_2),~\mathrm{where~}u_1,u_2\in U\right\} , \end{aligned}$$

under the control problem (23). The set *U* (control set) is such that

25 $$\begin{aligned} U&:= \big \{(u_1,u_2)|u_i(t)\, \text{ is } \text{ Lebesgue } \text{ measurable } \text{ on } ~[0,T],\nonumber \\ 0 &\le u_i(t)\le 1,~i=1,2\big \}. \end{aligned}$$

In addition, to discuss the existence analysis, the control problem can be expressed as

$$\begin{aligned} \frac{dY}{dt}=LY+N(Y), \end{aligned}$$

where

$$\begin{aligned} Y=\left( \begin{array}{c} S \\ A \\ B \\ R \end{array} \right) ,\quad L=\left( \begin{array}{cccc} -u_1(t)-\mu _0 &{} 0 &{} -\Lambda \eta &{} 0 \\ 0 &{} -u_2(t)-q_{2} &{} 0 &{} 0 \\ 0 &{} \beta &{} -\Lambda \eta -q_{3}-u_2(t) &{} 0 \\ u_1(t) &{} \gamma _1+u_2(t)&{} \gamma _2+u_2(t) &{} -\mu _0 \end{array} \right) , \end{aligned}$$

and

$$\begin{aligned} N(Y)=\left( \begin{array}{c} \Pi -\alpha SA-\gamma \alpha SB \\ \alpha SA+\gamma \alpha SB \\ 0 \\ 0 \end{array} \right) \end{aligned}$$

Let us define that $F(Y)=LY+N(Y)$, then for any $Y_1$ and $Y_2$, we have

$$\begin{aligned} \Vert F(Y_1)-F(Y_2)\Vert \le \mathcal {Q}\Vert Y_1-Y_2\Vert , \end{aligned}$$

where $\mathcal {Q}=\max \left\{ \mu _0,\mu _0+\mu _1\right\}$, known as the Lipschitz constants, and hence the function *F*(*Y*) is Lipschitz continuous, which ensures the existence of an optimal solution to the proposed control problem. Now to determine the existence of optimal controls, one has to prove their existence.

### Existence analysis

We now perform the existence analysis of the optimal controls for the proposed control problem as stated by the equations (22)–(25). For this, we prove that the set of control and associated states of the model are not empty as well as the control set is closed and convex. The state system of the control problem is linear in the control variables, while the integrand of the objective functional is convex over the control set *U*. Thus, regarding the existence analysis, we state the underlying result.

#### Theorem 6.1

For the control problem (22)–(25), there exists a pair of optimal values $u^{*}=(u_1^{*},u_2^{*})\in U$, such that

$$\begin{aligned} J(u^{*})=\min J(u_1,u_2). \end{aligned}$$

#### Proof

In order to discuss the existence of optimal controls, we use Theorem 9.2.1, p.182, given by Lukes in^44^, and followed by various authors^45,46^. Note that both the state and control variable of the model (23) have a non-negative value and bounded co-efficient, which implies that the control set and associated state variables are non-empty. Also, the solutions are bounded, and hence the control set is closed and convex. The state system of the control problem is linear in the control variables, implying that the optimal system is bounded. In addition, the integrand of the objective functional (22) is convex over *U*, therefore there exists a constants $\zeta >1$ and positive numbers $\xi _1$ and $\xi _2$ such that

$$\begin{aligned} J(u_1,u_2,u_3)\ge \xi _1+\xi _2\left( |u_1|^2+|u_2|^2\right) ^{\frac{\zeta }{2}}, \end{aligned}$$

which is enough for the optimal control pair existence. $\square$

### Optimality conditions

To examine an optimal solution to (22)–(25), we determine the Hamiltonian on the basis of Lagrangian by assuming that $x = \left( S, A, B, R\right)$ and $u = \left( u_1, u_2\right)$ be the vectors of state and control measure, respectively. Denoting the Lagrangian and Hamiltonian by *L* and *H* respectively, then one may write

26 $$\begin{aligned} L = w_1 A + w_2 B + w_3 u_1^2/2 + w_4 u_2^2/2 \end{aligned}$$

and

27 $$\begin{aligned} H = -L+\lambda \cdot g. \end{aligned}$$

In the above equations, we have taken $\lambda = \left( \lambda _1,\lambda _2,\lambda _3,\lambda _4\right)$ and $g = \left( g_1,g_2,g_3,g_4\right)$ where

28 $$\begin{aligned} \begin{aligned} g_1&= \left\{ 1-\eta B\right\} \Lambda -\alpha S A-\gamma \alpha S B-\left\{ u_1+\mu _0\right\} S,\\ g_2&= \alpha S A+\gamma \alpha SB-\left\{ \mu _0+\beta +\gamma _1\right\} A-u_2 A,\\ g_3&= \beta A -\left\{ \mu _0+\mu _1+\gamma _2-\Lambda \eta \right\} B-u_2 B,\\ g_4&= \gamma _2 B +\gamma _1 A+ u_1 S -\mu _0 R + u_2 \left\{ A + B\right\} . \end{aligned} \end{aligned}$$

We now find the solution (optimal) to the control model and exploit the conventional Maximum Principle^47^: Let $(x^{*},u^{*})$ represents the optimal solution, then one can find a non-trivial function $\lambda$ satisfying

29 $$\begin{aligned} \displaystyle \frac{dx^{*}}{dt} = \frac{\partial H}{\partial \lambda }(\lambda ,u^{*},x^{*}), \displaystyle \frac{d\lambda }{dt}= -\frac{\partial H}{\partial x}(\lambda ,u^{*},x^{*}), \end{aligned}$$

with the maximality condition

30 $$\begin{aligned} H(\lambda ,u^{*},x^{*}) = \max _{u \in [0,1] \times [0,1]} H(\lambda ,u^{*},x^{*}); \end{aligned}$$

and the transversality condition

31 $$\begin{aligned} \lambda = 0;~\text {whenever}~T=0, \end{aligned}$$

satisfied.

The next theorem follows from (26)–(31). We note that the optimality conditions given in^31^ are inconsistent. It is clear that in the case of minimization and a Hamiltonian, together with the associated multiplier as well as Lagrangian $+1$, the Pontryagin principle emphasizes a condition of minimality rather than a condition maximality. Here, condition (30) is the maximality condition for the proposed minimization problem as the multiplier $-1$ of the Hamiltonian associated with *L*.

#### Theorem 6.2

Assume $S^{*}$, $A^{*}$, $B^{*}$ and $R^{*}$ respectively denote the optimal state variables with the accompanying optimal measures $(u_1^{*},u_2^{*})$ for the problem (22)–(25), then $\lambda _i(t)$, $i = 1,\ldots , 4$ exists i.e., the adjoint variables exist which satisfy

32 $$\begin{aligned} \begin{aligned} \lambda _1^{'}(t)&= \left\{ \alpha A^{*}+\gamma \alpha B^{*}\right\} \left\{ \lambda _1(t)-\lambda _2(t)\right\} +\lambda _1(t)\left\{ \mu _0 + u_1^*\right\} -\lambda _4(t) u^{*}_1,\\ \lambda _2^{'}(t)&= w_1+\left\{ \lambda _1(t)-\lambda _2(t)\right\} \alpha S^{*}+\lambda _2(t)\mu _0-\left\{ \lambda _4(t)-\lambda _2(t)\right\} \gamma _1\\&-\left\{ \lambda _4(t)-\lambda _2(t)\right\} u^{*}_2 +\left\{ \lambda _2(t)-\lambda _3(t)\right\} \beta ,\\ \lambda _3^{'}(t)&=w_2-\left\{ \lambda _2(t)-\lambda _1(t)\right\} \gamma \alpha S^{*}-\left\{ \lambda _3(t)-\lambda _1(t)\right\} \Lambda \eta \\&-\left\{ \lambda _4(t)-\lambda _3(t)\right\} \left\{ u^{*}_2+\gamma _2\right\} +\left\{ \mu _1+\mu _0\right\} \lambda _3(t),\\ \lambda _4^{'}(t)&=\mu _0\lambda _4(t). \end{aligned} \end{aligned}$$

The terminal (transversality) conditions associated are

33 $$\begin{aligned} \lambda _i=0,~\text {whenever}~T=0. \end{aligned}$$

The optimal measures $u_1^{*}$ and $u_2^{*}$ are as

34 $$\begin{aligned} u_1^{*} =\max \left\{ \min \left\{ \frac{1}{w_3}S^{*}(\lambda _4-\lambda _1), 1\right\} ,0\right\} , \end{aligned}$$

and

35 $$\begin{aligned} u_2^{*} =\max \left\{ \min \left\{ \frac{1}{w_4}(\lambda _4-\lambda _3)B^{*}-(\lambda _2-\lambda _4)A^{*},1\right\} ,0\right\} . \end{aligned}$$

#### Proof

System (32) is derived from the Pontryagin Maximum Principle i.e. from the 2nd relation of (??) with the Hamiltonian as described in (26)–(28), while conditions (33) follow from the transversality condition (31). To derive $u^{*}_1$ and $u^{*}_2$, we differentiate the Hamiltonian partially and solve $\frac{\partial H}{\partial u_1}=0$ and $\frac{\partial H}{\partial u_2}=0$ for control measures. Finally, with the help of the maximality condition (30), we derive (34)–(35). $\square$

We find the optimal measures by investigating the optimal system (23) and the adjoint (32), along with boundary conditions and (33), along with $(u^{*}_1,u^{*}_2)$ given by (34) and (35) numerically.

## Numerical simulations

To support our theoretical results, we present the numerical investigations using the numerical procedure of Runge-Kutta method of the 4th order. Parameters for a disease-free state are taken as follows:

36 $$\begin{aligned} \Lambda &= 0.0121,\ \eta =0.8,\ \mu _0=0.00693,\ v = 0.002,\ \alpha = 0.95,\\ \gamma &= 0.16,\ \gamma _1 = 0.004,\ \beta =0.33,\ \gamma _2=0.002,\ \mu _1=0.8. \end{aligned}$$

**Figure 2:**
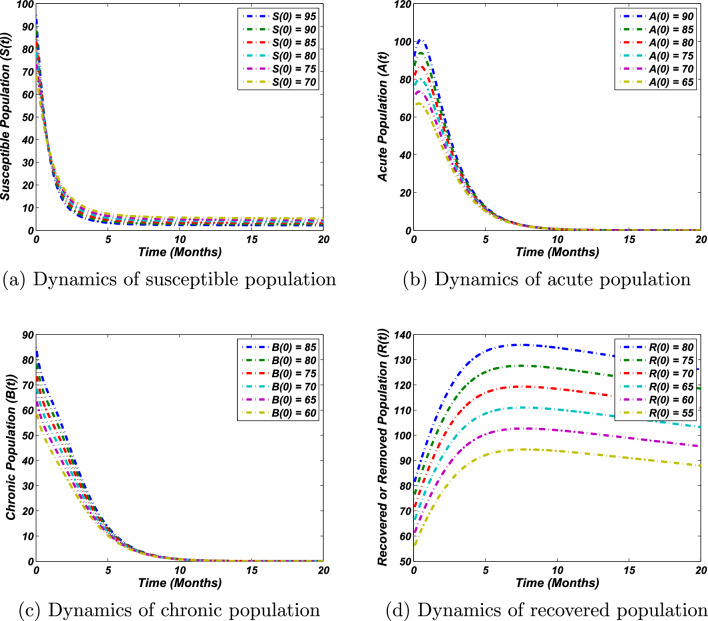
Solution curves of the system (1) around the disease free equilibrium against the parameters value given in Eq. (36) and for different initial sizes of population, where the value of threshold quantity (basic reproductive number), $R_0= 0.203<1$.

We have taken some parameter values from the literature. In addition, some are assumed with feasible values based on sufficient analysis and calculation of the conditions that satisfy the stability results. In particular, $\Lambda$, $\mu _0$, $\beta$, $\gamma$ and $\gamma _2$ are taken from^3^, while all other parameters are assumed. Clearly, in this case, the proposed problem, as stated by equation (1), has only the infection-free state and is stable globally asymptotically (see Fig. 2). Note that for these values, the calculation of the basic reproductive number gives that $R_0=0.203$ implies that $R_0<1$, so the stability results at the disease-free states hold. In addition, the theoretical interpretation states that if $R_0<1$, each solution curve of *S* approximately taking five months to reach its associated equilibrium position as depicted in Fig. 2a. Similarly, the dynamics of the acute and chronic population are demonstrated in Fig. 2b,c, which describe that the solution curves of *A* and *B* take approximately ten months to reach the stable equilibrium position. On the other hand, the dynamics of the recovered population reveal that there will always be recovered individuals, as presented by Fig. 2d. Thus biologically, the results state that eliminating the hepatitis B virus from the community is subject to the threshold parameter’s value. Whenever it is less than unity, the disease will be easily eradicated.

**Figure 3:**
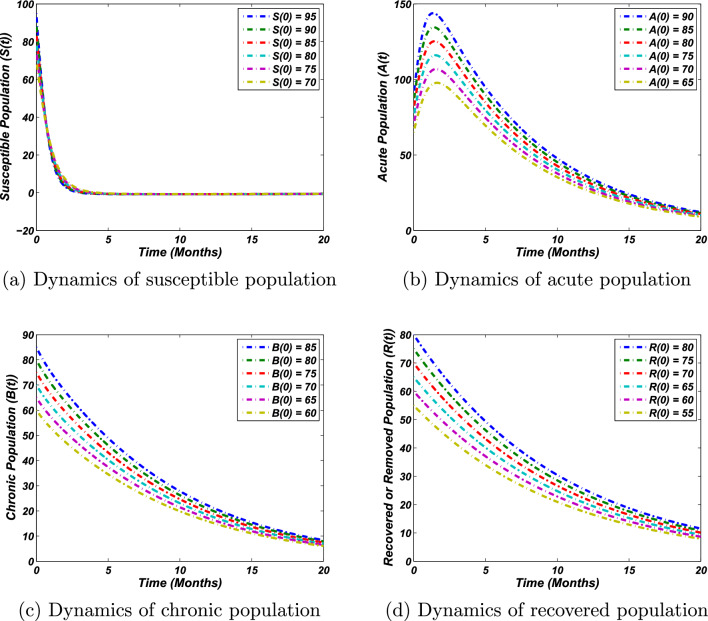
Solution curves of system (1) around the endemic equilibrium against the parameters values given in Eq. (36), except $\alpha =0.95$ and different initial sizes of population, which implies that $R_0 = 2.03>1$.

For investigating the stability of the proposed epidemiological model, we assume the same parameter values as in Eq. (36), except for $\alpha .$. If $\alpha =0.95$, then $R_0=2.03>1$ implies that the endemic state exists, as illustrated in Fig. 3, which clearly shows the results of the analytical analysis.. In this case, the biological investigations reveal that if no control measures are adopted appropriately, the disease will reach its associated endemic position. It could be noted that the susceptible portion of the population decreases from the initial and leads to zero over time, as shown in Fig. 3a. However, there will always be an infected population, i.e., chronic and acute individuals, as shown in Fig. 3b,c, respectively. Similarly, we simulate the problem to study the dynamics of the recovered population as illustrated in Fig. 3d. The time dynamics of the recovered population state that the amount of recovered individuals decreases as time grows while leading to its associated endemic position.

We now perform the numerical investigation of the control problem to verify and support the theoretical examinations for optimal control analysis. We again use the 4th-order Runge-Kutta (RK) technique to perform the numerical simulations of the control problem. More precisely, we solve the system (23) via the 4th order RK scheme by taking the time unit from 0 to 50. We then solve the adjoint variables system as given by Eq. (32) with the help of backward RK procedure of the 4th order at the same interval of time along with the use of the transversality conditions stated by Eq. (33) as well as with the solution of the state system. To investigate the endemic state of the model, we use the same parametric values. The weight constants and initial conditions are, however, as follows:

37 $$\begin{aligned} \begin{aligned} w_1&=0.10,\quad w_2=0.6,\quad w_3=0.001,\quad w_4=0.9,\\ S(0)&=20,\quad A(0)=10,\quad B(0)=10,\quad R(0)=10. \end{aligned} \end{aligned}$$

**Figure 4:**
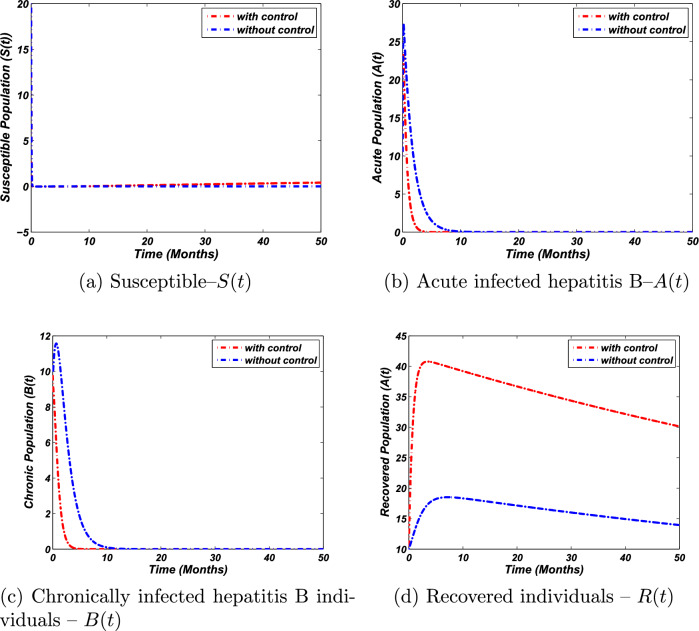
The temporal dynamics of the model with optimal control verse without control ($v = 0.02$) against the parametric values (37).

**Figure 5:**
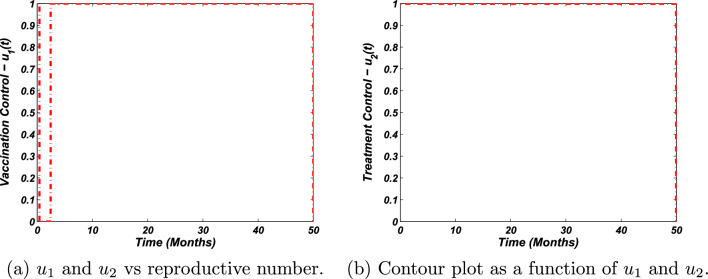
The plot represents the influence of the control measures on the basic reproductive number.

We then execute the above procedure with the aid of Matlab and obtain the graphical visualization as presented in Fig. 4, demonstrating the time dynamics of epidemiological groups of susceptible, acute, chronic, and recovered individuals. Our numerical results illustrate clearly the effect of applying the controls: to minimize acute and chronically infected populations while maximizing the recovered individuals. The illustration of the susceptible population with and without control is described in Fig. 4a. Moreover, Fig. 4b depicts the acute population with and without control. In a similar fashion, the dynamics of a chronic population with and without controls are shown in Fig. 4c. Finally, Fig. 4d visualizes the simulation of the recovered population with the application and without the application of controls. Further, Fig. 5 presents the control profiles. These analyses clearly, reflect the importance and the effectiveness of the implantation of the proposed control mechanism.

We note that the numerical simulations presented in^31^, aiming to illustrate the usefulness of optimal control theory, are inconsistent. Indeed, in^31^, the authors solved an inconsistent control problem using two control measures: isolation and treatment. However, in the case of hepatitis B, where more than two billion people are infected, the control isolation is inconsistent according to the WHO guideline and is never exercised.

**Figure 6:**
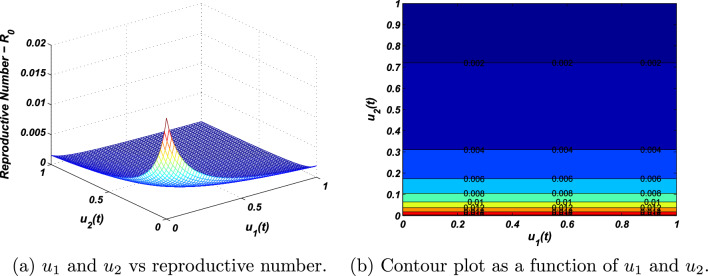
The plot represents the influence of the control measures on the basic reproductive number.

Additionally, we observe that the reproductive number is a key parameter, and when this quantity is greater than unity, the disease persists, while if it is less than one, it becomes extinct. Using the basic reproduction numbers as a reference, we conduct a sensitivity analysis of the model parameters against the control measures. Some parameters are directly proportional to the reproductive number, while others are negatively proportional. Vaccination and treatment controls are negatively correlated with reproductive quantity, such that whenever their values increase, the reproductive number decreases significantly, as shown in Fig. 6. Based on the parametric values given in Eq. (36), the sensitivity indexes of the vaccination and treatment control measures are calculated. It can be seen in Fig. 6a,b that if the control measures were increased by 10 percent, the reproductive number would decrease by 7.14 percent. Hence, we conclude that implementing the proposed control strategies in a true sense will result in the eradication of contagious hepatitis B virus infections.

## Conclusions

Our study examined the dynamics of hepatitis B epidemics under acute and chronic transmission scenarios, both horizontally and vertically. After mathematically deriving and analyzing the proposed system, we determined its reproduction number to find the model equilibrium and stability. In the proposed problem, there are two equilibrium states: infection-free and disease-endemic. There is a detailed description of both steady states. Under certain conditions, the equilibria are stable. The global properties of the proposed epidemiological model were analyzed using the Lyapunov function theory and the geometrical approach. Additionally, we demonstrated that the proposed problem exhibits backward bifurcation. To develop an optimal control mechanism, the proposed problem incorporates two time-dependent control measures, vaccination, and treatment. According to Pontryagin’s necessary conditions, there exists an optimal control mechanism for minimizing the infected (acute and chronic). We concluded by presenting numerical justifications and examining whether the derived analytical findings are robust. Treatment and vaccination, along with the application of optimization theory, were found to be very effective in controlling hepatitis B virus infection. Accordingly, we do not recommend the isolation of individuals, as opposed to the results reported in^31^. Based on different perspectives of the results, which were investigated analytically and numerically, we concluded that one way to eradicate hepatitis B is to minimize the threshold quantity by keeping it below unity. The model also suggests that if hepatitis B persists, it will reach its endemic status, which is high and dangerous for the community. As a result, various control strategies must be utilized to prevent the spread of the disease. Vaccination and treatments are used as time-dependent controls, demonstrating a significant impact on the control of hepatitis B virus transmission.

Although the work reported in this research yielded interesting results, we will discuss both the singular and nonsingular fractional versions of the model in our future publication to obtain more accurate dynamics.

### Ethical approval

All the authors demonstrating that they have adhered to the accepted ethical standards of a genuine research study.

### Consent to participate

Being the corresponding author, I have consent to participate of all the authors in this research work.

## Data Availability

Data will be provided on request to the corresponding author.
